# Determination of Flavonoid Compounds in Shanxi Aged Vinegars Based on Hydrophobic Deep Eutectic Solvent VALLME-HPLC Method: Assessment of the Environmental Impact of the Developed Method

**DOI:** 10.3390/molecules28145619

**Published:** 2023-07-24

**Authors:** Baoqing Bai, Yanli Guo, Siyuan Meng, Shujun Chen, Tao Bo, Jinhua Zhang, Dan Shen, Yifei Liu, Yukun Yang, Sanhong Fan

**Affiliations:** 1 School of Life Science, Shanxi University, Taiyuan 030006, China; baoqingbai@sxu.edu.cn (B.B.); gyl05182022@163.com (Y.G.); 202223109012@email.sxu.edu.cn (S.M.); chensj@sxu.edu.cn (S.C.); botao@sxu.edu.cn (T.B.); ever840605@sxu.edu.cn (J.Z.); 202123117011@email.sxu.edu.cn (D.S.); ydcamille@163.com (Y.L.); 2 Shanxi Key Laboratory for Research and Development of Regional Plants, Shanxi University, Taiyuan 030006, China; 3 Key Laboratory of Chemical Biology and Molecular Engineering of Ministry of Education, Institute of Biotechnology, Shanxi University, Taiyuan 030006, China

**Keywords:** Shanxi aged vinegar, hydrophobic deep eutectic solvent, vortex-assisted liquid–liquid microextraction, flavonoid, green analysis

## Abstract

This research presents a novel, eco-friendly, vortex-assisted liquid–liquid microextraction (VALLME) approach, integrating hydrophobic deep eutectic solvents (DESs) with HPLC for the identification and quantification of nine specific flavonoids in Shanxi aged vinegar (SAV). The parameters of DES-VALLME, including the ratio of trioctylmethylammonium chloride to 1,4-butanediol (1:6), DES volume (150 μL), vortex duration (5 min), the concentration of NaCl (0.40 g), and centrifugation time (10 min), were optimized to achieve the maximum extraction efficiency of target substances. Under these optimal conditions, quantitative analyses performed via HPLC demonstrated a broad linear range of 0.20–50.00 μg/mL and correlation coefficients (r^2^) greater than 0.9944 for all nine calibration curves. The limits of detection (LOD) and limits of quantitation (LOQ) were 0.09–0.18 μg/mL and 0.30–0.60 μg/mL, respectively, ensuring high sensitivity. The relative standard deviations for intra-day and inter-day variability were within the acceptable range, 2.34–3.77% and 3.04–4.96%, respectively, demonstrating the method’s reliability. The recovery rates ranged from 85.97% to 108.11%, underscoring the method’s precision. This technique exhibited a significant enrichment effect (enrichment factor: 43 to 296) on SAV flavonoids. Notably, the eco-friendliness of this procedure was evaluated using the Analytical Eco-Scale, Green Analytical Procedure Index, and Analytical Greenness Metric. The results suggested that this technique is a viable green alternative to traditional flavonoid determination methods in SAV. In summary, this novel method provides a theoretical basis for assessing flavonoid content in SAV samples and tracing SAV products. This contribution has significant implications for enhancing analytical techniques in food chemistry and environmental science and the sustainable development of the food industry.

## 1. Introduction

Vinegar, a ubiquitous acidic condiment, is cherished globally for its ability to impart a unique tartness to foods [1]. In China, the main raw materials for vinegar production include sorghum, millet, glutinous rice and other grains. Prominent among these are Shanxi aged vinegar (SAV), Zhenjiang aromatic vinegar (ZAV), Sichuan bran vinegar (SBV) and Fujian Monascus vinegar (FMV). Among these, SAV holds a special place as one of the four traditional fermented vinegars in China. It is renowned for its nutritional richness, including amino acids, organic acids, sugars, vitamins, ligustrazine, total flavonoids and a myriad of volatile flavor substances [2]. Recent medical research has highlighted the high antioxidant properties of flavonoids, a group of bioactive ingredients found in relatively high concentrations in SAV [3]. In addition to their antioxidant properties, flavonoids exhibit a broad spectrum of health-promoting effects, such as antimicrobial activity [4], immune system enhancement [5], anticancer properties [6] and cholesterol-lowering effects [7]. Despite their crucial role in health and nutrition, most studies on flavonoids in SAV have primarily been limited to determining total flavonoids [8]. However, the comprehensive quantitative analysis of flavonoids with high content in SAV has not been extensively explored.

At present, a variety of methods are utilized to identify flavonoids, including ultraviolet-visible (UV-Vis) spectrophotometry [9], thin layer chromatography (TLC) [10], nuclear magnetic resonance spectroscopy (NMR) [11] and high performance liquid chromatography (HPLC). However, these procedures are often hampered by the complex matrices involved, making the direct quantification of flavonoids in actual samples a time-intensive process [12]. Therefore, it is necessary to prepare samples before instrumental analysis [13]. Conventional sample preparation methods encompass solvent extraction [14], microwave-assisted extraction [15], ultrasonic-assisted extraction [16,17], supercritical extraction [18,19] and the resin method [20]. Traditional solvent extraction, though effective, involves the extensive use of organic solvents during extraction, leading to notable environmental concerns. From the perspective of green analytical chemistry (GAC), it is crucial to minimize the utilization of organic solvents to reduce their environmental footprint. In recent years, the vortex-assisted liquid–liquid microextraction (VALLME) method has gained increased attention from researchers due to its numerous advantages, including reduced extraction time, lower solvent consumption and energy efficiency [21]. As a result, this method has found broad applications in analytical chemistry research. Nonetheless, selecting appropriate dispersion and extraction solvents remains a significant challenge. Conventional liquid–liquid extraction methods often use relatively toxic organic solvents, such as chloroform, carbon tetrachloride and chlorobenzene [22]. These solvents pose potential threats to both human health and the environment.

Deep eutectic solvents (DESs) have recently gained substantial interest as an eco-friendly substitute for traditional organic solvents. These solvents are synthesized via the interaction of organic compounds and ionic compounds via hydrogen bonds [23]. They are characterized by their environmentally friendliness, biodegradability, low vapor pressure, high conductivity and stable chemical properties [24]. In recent years, the expanding field of material science has further broadened the application prospects of DESs. Indeed, they have become a focal point of analytical chemistry research. For instance, Funda et al. [25] developed a novel DES-LLME method by employing choline chloride as a hydrogen bond acceptor and phenol as a hydrogen bond donor. They successfully used this method, in conjunction with a UV-Vis spectrophotometer to analyze and determine the target analytes, to separate and preconcentrate curcumin in food and herbal tea samples. Zhu et al. [26] proposed a novel technique for determining quantities of eight synthetic pigments in beverage samples by utilizing hydrophobic DES as microextraction solvent in LLME coupled with HPLC.

In this work, a novel approach was devised for the extraction of flavonoids from SAV by employing hydrophobic deep eutectic solvents in combination with vortex-assisted liquid–liquid microextraction and high-performance liquid chromatography (DES-VALLME-HPLC). Not only were the key parameters influencing the extraction efficiency thoroughly examined, but the method also demonstrated the capability to rapidly and efficiently analyze the contents of nine flavonoids across 40 SAV samples. Additionally, the “greenness” or eco-friendliness of the DES-VALLME-HPLC method was assessed using three specific evaluation tools: the Analytical Eco-Scale, Green Analytical Procedure Index and Analytical Greenness Metric. This comprehensive evaluation underscores our commitment to developing greener methodologies in analytical chemistry in line with the broader scientific community’s increasing focus on sustainable practices.

## 2. Results and Discussion

### 2.1. Characterization

**Figure 1:**
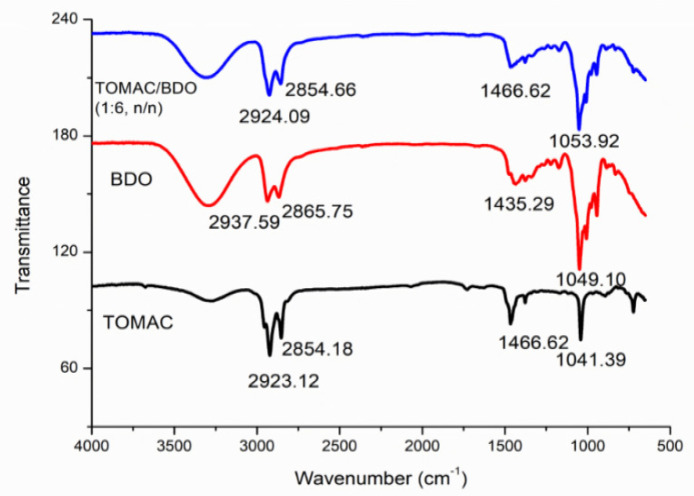
FT–IR spectra of tricaprylylmethylammonium chloride, 1,4-butanediol and DES5.

The Fourier transform infrared (FT−IR) spectra of trioctylmethylammonium chloride, 1,4-butanediol and DES5 are shown in Figure 1. The C-H stretching vibrations observed within the range of 2845–3007 cm^−1^ are attributable to an alkane found between the methyl and methylene groups [27]. The -CH_2_ in trioctylmethylammonium chloride peaked at 1466.62 cm^−1^ with a pronounced and sharp peak. However, the corresponding peak in DES5 exhibited a broader shape and diminished intensity at the same wavenumber, indicating a significant transformation. Moreover, C-OH absorption in 1,4-butanediol was prominently visible at 1049.10 cm^−1^, exhibiting a sharp peak with pronounced intensity. However, in the DES5 spectrum, the C-OH peak was recorded at a slightly higher wavenumber, 1053.92 cm^−1^, suggesting a redshift of the C-OH peak. This shift could be attributed to changes in the force constant prompted by a reduction in electron cloud density. This modification, in turn, reduces the stretching vibration frequency and substantially shifts the absorption peak of the proton donor towards a lower position [28]. The FT−IR data collectively validated the successful formation of hydrogen bonds in DES.

### 2.2. Optimization of the Extraction Procedure

#### 2.2.1. Effect of the Types of DES

**Figure 2:**
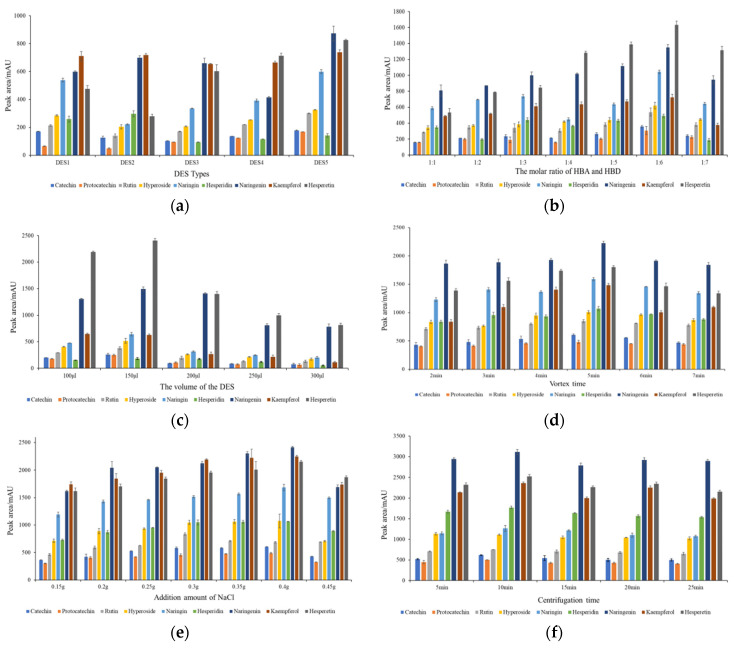
The results of the effect of the types of DES (**a**), the molar ratio of HBA and HBD (**b**), the volume of the DES (**c**), vortex time (**d**), the addition amount of NaCl (**e**) and centrifugation time (**f**).

In this study, we examined five distinct types of deep eutectic solvents (DESs) (Figure 2a). Among these, the tricaprylylmethylammonium chloride served as the HBA. At the same time, n-caprylic acid, ethylene glycol, 2,3-butanediol, 1,3-butanediol and 1,4-butanediol performed the role of HBD. Our findings indicated that DES5 extracted the highest peak areas for catechin protocatechin, rutin, hyperoside, naringin, naringenin, kaempferol and hesperetin. This likely occurred because of the fact that DES5 has the lowest viscosity and is more highly permeable to the pores in the matrix [29]. Based on these results, DES5 was selected as an extraction solvent for the subsequent procedures due to its superior extraction performance.

#### 2.2.2. Effect of the Molar Ratio of HBA and HBD

The extraction efficiency of the target compounds is significantly influenced by the molar ratio of the DES. Observations indicated that as the molar ratio of 1,4-butanediol increases from 1:1 to 1:6, the peak areas of the target compounds progressively increase. The peak areas reached their maximum value when the molar ratio was 1:6. Upon further increasing the molar ratio, the peak areas started to decline (Figure 2b). The phenomenon can be postulated to occur due to the enhanced dispersibility of DESs in water with the addition of 1,4-butanediol, thus enhancing the peak area [30]. Besides that, the molar ratio of DES influences the viscosity of DESs, which, in turn, affects the mass transfer rate [31]. During this experiment, it was noted that tricaprylylmethylammonium chloride exhibited greater viscosity compared to 1,4-butanediol. Consequently, as the proportion of 1,4-butanediol increased, the DES’s viscosity gradually diminished, presumably boosting the extraction effect of the DES [32]. In summary, the molar ratio of tricaprylylmethylammonium chloride and 1,4-butanediol was determined to be 1:6.

#### 2.2.3. Effect of the Volume of the DES

As shown in Figure 2c, the peak area of the target compounds increased when the volume of DES5 increased from 100 μL to 150 μL. This surge can likely be attributed to the previous inadequacy of the target compound’s extraction due to the limited volume of DES5 [22]. Upon the DES5 reaching a volume of 150 μL, the peak areas reached their maximum, suggesting that an equilibrium between the volume of DES5 and the extraction effect had been achieved [33]. Conversely, when the volume of DES5 exceeded 150 μL, a decline in the peak areas was observed. This reduction may have resulted from the excessive volume of the extractant, causing a decrease in the enrichment effect [34]. Therefore, considering both the extraction efficacy and the economic implications, a volume of 150 μL was selected as the optimal volume for DES5.

#### 2.2.4. Effect of Vortex Time

Figure 2d illustrates a notable trend in the peak areas of the targets, which initially increased and then decreased as the vortex time varied from 2 min to 7 min. A positive correlation was observed between the peak areas and vortex duration when the former was between 2 and 5 min. This likely occurred because an extended extraction period facilitates a higher yield of analytes into the extractant [35]. However, when the vortex time was extended from 5 to 7 min, the peak area negatively correlated with the vortex duration. The likely explanation is that excessively long extraction times may lead to a loss of extractant, thereby reducing extraction efficiency [36]. In light of these observations, we selected a vortex duration of 5 min for further investigation.

#### 2.2.5. Effect of the Addition Amount of NaCl

The amount of NaCl added is another parameter that influences the extraction efficiency of the analyte. The salting-out effect can reduce the solubility of the target in the sample solution, facilitating the isolation of target molecules [37]. As shown in Figure 2e, the peak area gradually increased when the NaCl content was increased from 0.15 g to 0.40 g. This is because the ion concentration in the sample solution increased, decreasing the solubility of the target substance and promoting its transfer to the extractant solution [38]. However, when the NaCl content further increased from 0.40 to 0.45 g, the peak areas of the target compounds decreased. This may be due to the fact that when the solution reaches saturation at a NaCl content of 0.40 g, continued addition of salt increases the viscosity and density of the solution, which hinders the transfer of the target from the aqueous matrix to the extraction solvent [35]. Therefore, the optimal amount of NaCl was determined to be 0.40 g.

#### 2.2.6. Effect of Centrifugation Time

The selection of centrifugation time also plays a crucial role in the extraction process by eliminating emulsification via high-speed centrifugal separation, effectively separating the organic phase from the aqueous phase [39]. In this study, we examined five levels of centrifugation time (5 min, 10 min, 15 min, 20 min and 25 min). Our results showed that increasing the centrifugation time from 5 to 10 min slightly increased extraction efficiency. However, further increases in centrifugation time beyond 10 min did not result in significant changes in extraction efficiency (Figure 2f). Based on these findings, we set the optimal centrifugation time at 10 min.

### 2.3. Analysis of Box-Behnken Design Results

Response surface design was constructed based on the above single factor test using Design-Expert 8.0.6. This experiment focused on exploring four key factors: the volume of the DES (A), vortex time (B), addition amount of NaCl (C) and centrifugation time (D). Each factor was assigned three levels, designated as high level (1), middle level (0) and low level (−1). The levels of every factor were shown in Table 1.

After analyzing 29 groups of experiments, the fitted quadratic multiple regression equations were as follows,

Y_1_ = 569.38 + 54.35A − 12.06B − 5.43C − 12.09D − 9.38AB + 13.30AC + 4.37AD − 53.47BC − 4.58BD + 1.13CD − 170.26A^2^ − 57.22B^2^ − 45.04C^2^ − 77.40D^2^;

Y_2_ = 537.88 + 40.94A − 8.85B − 19.39C − 16.69 − 6.63AB + 22.23AC + 8.97AD − 24.74BC − 3.45BD + 16.10CD − 167.31A^2^ − 51.62B^2^ − 10.97C^2^ − 63.84D^2^;

Y_3_ = 810.80 + 72.26A − 20.95 − 20.50C − 18.99D − 29.50AB + 32.90AC + 2.34AD − 30.88BC + 0.31BD + 27.71CD − 263.43A^2^ − 54.80B^2^ − 8.11C^2^ − 46.48D^2^;

Y_4_ = 1116.56 + 105.48A − 66.74B − 49.18C + 0.27D + 4.60AB − 25.30AC + 14.90AD − 85.87BC − 42.45BD + 8.24CD − 363.04A^2^ − 77.50B^2^ − 31.62C^2^ − 43.90D^2^;

Y_5_ = 1106.36 + 101.41A − 64.70B − 51.18C + 1.21D + 4.34AB − 23.23AC + 14.76AD − 87.09BC − 41.90BD + 8.33CD − 356.14A^2^ − 78.98B^2^ − 30.19C^2^ − 48.19D^2^;

Y_6_ = 1277.22 − 156.08A + 12.92B + 4.70C − 14.10D − 59.90AB + 1.15AC + 33.88AD − 60.85BC + 6.11BD − 70.02CD − 171.07A^2^ − 81.47B^2^ + 38.76C^2^ − 169.47D^2^;

Y_7_ = 2767.68 − 416.75A − 152.45B + 26.91C − 126.58D + 76.95AB + 13.76AC − 107.30AD − 169.24BC + 148.01BD + 333.61CD − 266.94A^2^ − 390.86B^2^ − 134.38C^2^ − 145.99D^2^;

Y_8_ = 2065.82 − 286.61A + 102.73B − 95.16C − 80.41D + 34.85AB−95.53AC + 375.59AD − 7.68BC − 59.54BD − 11.73CD − 428.40A^2^ − 458.51B^2^ − 390.12C^2^ − 41.32D^2^;

Y_9_ = 2567.10 − 363.00A + 92.03B + 422.19C + 18.26D + 29.18AB − 27.15AC + 91.23AD − 236.62BC − 36.95BD − 67.85CD − 550.05A^2^ − 281.97B^2^ − 461.11C^2^ − 1379.23D^2^.

In the above equations, Y_1_, Y_2_, Y_3_, Y_4_, Y_5_, Y_6_, Y_7_, Y_8_ and Y_9_ represented the peak areas of catechin, protocatechin, rutin, hyperside, naringin, hesperidin, naringenin, kaempferol and hesperetin. According to Table 2, the relative importance of the four factors varied among these nine flavonoids in descending order and was as follows: A > D > B > C, A > C > D > B, A > B > C > D, A > B > C > D, A > D > B > C, A > D > B > C, A > B > D > C, A > B > C > D and C > A > B > D.

**Figure 3:**
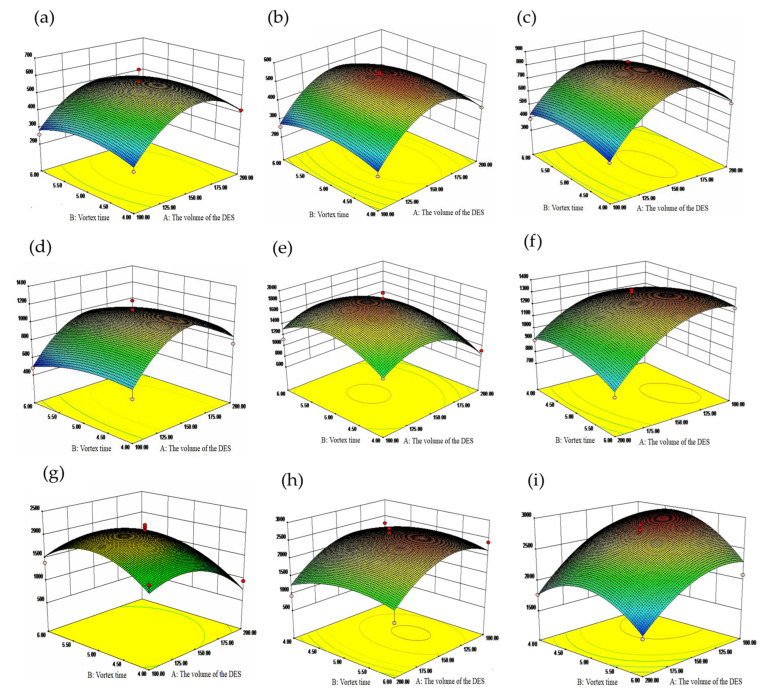
(**a**–**i**) Response surface plots for the influence of the volume of the DES and vortex time on the peak areas of the catechin, protocatechin, rutin, hyperside, naringin, hesperidin, naringenin, kaempferol and hesperetin.

Figure 3a–i depicts the response surface plots’ interplay between the volume of the DES (A) and the vortex time (B) on the peak areas of nine flavonoids. When the slope of the response surface was steep and the contour line was elliptical, a significant interaction between the two factors was suggested. On the other hand, a gentle slope with a contour line leaning towards a circular shape indicated a less significant interaction. The steep changing trend in Figure 3 indicated a significant interaction between the volume of the DES and vortex time.

### 2.4. Method Validation

The experimental analysis confirmed that the concentration range of each flavonoid, from 0.20–50.00 μg/mL, exhibited correlation coefficients between 0.9944–0.9990. This strong correlation suggests that the linear equation fits the data well. Then, an extensive set of tests were executed to validate this newly proposed method further, yielding interesting findings as elucidated below. The observed enrichment factors varied between 43 and 296, while the limits of detection (LODs) ranged from 0.09 μg/mL to 0.18 μg/mL. The limits of quantitation (LOQs) were established within the scope of 0.30 μg/mL to 0.60 μg/mL. Regarding precision, RSD values ranged between 2.34 and 3.77% for intra-day precision and between 3.04 to 4.96% for inter-day (n = 3) precision (Table 3). The ranges of recovery rate were 85.97–108.11%. When the spiked concentration of 9 flavonoids was set at 20 μg/mL, the spiked recoveries of 9 flavonoids in real vinegar samples (W3, D3, C2, YN1 and Z3) fluctuated between 85.97% and 108.11% (Table 4). Therefore, the method had good reliability and could be used for the subsequent determination of real samples.

### 2.5. Targeted Metabolomic Analysis of Flavonoids in Actual Vinegar Samples

**Figure 4:**
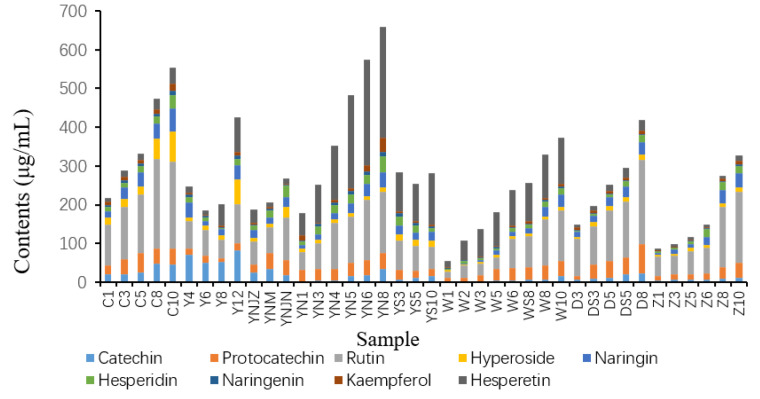
The contents of nine flavonoids in actual samples.

The composition of 9 flavonoids in 40 vinegar samples of 5 brands was analyzed. The examined flavonoids included catechin, protocatechin, rutin, hyperin, naringin, hesperidin, hesperidin, kaempferol and hesperidin. Variations in the dominant flavonoids among the test samples could be attributed to the dissimilarities in raw materials, acidity levels and production processes (Figure 4). Insights into brand-specific trends showed increased flavonoid contents corresponding with the rise in Shanxi aged vinegar’s (SAV) aging time. Brands Y and C, notably, demonstrated higher flavonoid content than the other brands. A comparative analysis between Brands Y and C revealed a similar concentration of catechin, procatechin and naringenin. However, Brand C exhibited a clear dominance in rutin, hyperoside and naringin concentrations over Brand Y. In contrast, the content of hesperidin, hesperetin and kaempferol was markedly elevated in Brand Y compared to Brand C. Further, the content of hesperetin was the highest in brand Y, followed by brand D, with significantly higher levels than the remaining brands. With the progressive increase in SAV’s aging years, a concurrent rise in hesperetin content was recorded across all five brands. Similarly, brand C displayed the highest rutin content, which was also relatively high in the other four brands. A decreasing trend in rutin content was noticed with the reduction in brand C’s ageing year, a pattern also reflected in the other brands. The graphical representation via column histograms (Figure 4) highlighted the peak flavone content in YN8 at approximately 657.95 μg/mL. Compared with other samples, the W1 sample demonstrated significantly lower total flavonoid content, measuring 54.77 μg/mL. As an individual flavonoid, rutin exhibited exceptionally high content across all samples, ranging between 16.32 μg/mL to 232.068 μg/mL.

### 2.6. Heat Map Analysis

**Figure 5:**
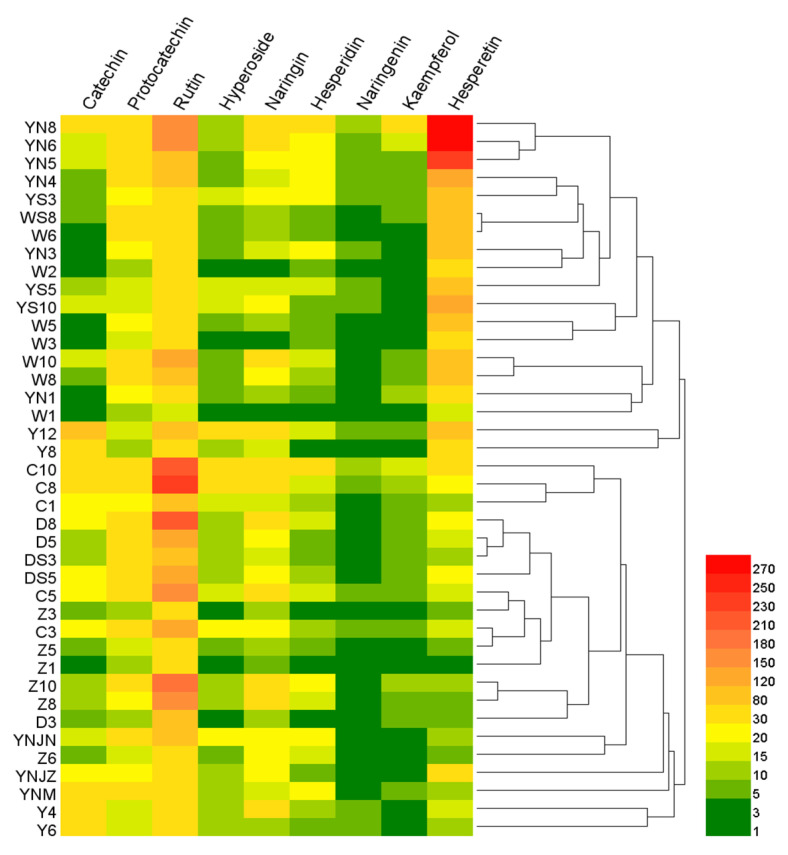
Hierarchical cluster analysis (HCA) of tested samples.

In Figure 5, a comparison of the contents of nine flavonoids in 40 samples is presented using a heat map. Each row within the graphic denotes a unique sample of SAV, and each column corresponds to a distinct flavonoid compound. High and low levels are depicted by red and green hues, respectively. The heat map facilitates a detailed analysis of the variations in flavonoid compounds across different brands and maturity levels of vinegar samples. The significant clustering suggests a high degree of similarity in flavonoid expression patterns amongst specific samples. Notably, the YN8, YN6 and YN5 samples displayed pronounced clustering, signifying an exceptionally high hesperetin content. Likewise, samples C10 and C8 manifested a distinct clustering pattern with a notably elevated rutin content. Upon close examination of Figure 5, it was observed that the concentrations of naringenin and kaempferol were generally lower across all samples. Broadly, samples from the same brand tended to cluster together, implying a relative consistency in the expression characteristics of flavonoids within each brand. This observation further substantiated the hypothesis that the characteristic flavonoids in a sample are influenced by the raw materials used, the processing methods employed and the sample’s acidity.

### 2.7. Assessment of Method’s Environmental Impact

In order to assess the environmental sustainability of the method developed in this study, three distinct evaluation metrics were employed. The first of these is the Analytical Ecology Scale (AES), which analyzes the environmental impact of the analytical process based on the assignment of penalty points (PPs) to various parameters. This method system principally penalizes the usage of harmful reagents, instruments and waste. The penalty associated with a reagent is determined by its hazard and quantity, with the penalty points calculated as the product of these two factors [40]. The hazard value of the reagent, in turn, is computed as the product of the number of associated hazard pictograms and signal words. The final score is determined by subtracting the total penalty points from 100, with scores ≥75, ≥50 and <50 corresponding to ‘excellent’, ‘acceptable’ and ‘inadequate’ environmental impact, respectively. In the context of this study, the total penalty points amounted to 20, yielding an AES score of 80. This score indicates that the method developed in this study has a commendable environmental profile (Table 5).

The Green Analytical Procedure Index (GAPI) constitutes the second evaluation metric used in this study. It comprehensively assesses the entire analytical process from sample collection to final determination. The GAPI encompasses five key aspects: sample collection, sample preparation, solvents and reagents, instrumentation use and waste generation. The environmental impact of each stage is visually represented via a three-tiered color-coded pictogram system, spanning from green (low environmental impact) to red (high environmental impact) [41]. As depicted in Table 6, our method is predominantly represented by green sections (six in total), with only five red sections. This pictogram indicates that our method is environmentally friendly, posing minimal hazards to human health and the environment.

The third and final assessment tool is the Analytical Greenness Metric (AGREE). This metric evaluates the environmental sustainability of an analytical method by deploying a set of tools grounded in the 12 principles of Green Analytical Chemistry) [42]. The environmental friendliness of a method is quantified on a scale from 0 to 1 using pictograms. Comparing our approach with similar recent reports (Table 7). Due to the lack of information about SAV, we chose to have the report focus on extraction flavonoids. Indeed, the proposed procedure is the greenest methodology (0.71 versus 0.59, 0.5 and 0.6) due to the replacement of organic solvents such as 60% ethanol, CHCl_3_ or chloroform with DES (TOMAC: BDO). Sample usages and extractant volumes also lead to better and more sustainable analytical methods. Therefore, the method can be classified as environmentally friendly. The amalgamation of the above three evaluation tools provides a holistic assessment of our method’s overall environmental impact, encompassing all stages from sample preparation to chromatographic analysis [43].

### 2.8. Comparison of the Optimized Method with Other Procedures

The developed hydrophobic DES-VALLME-HPLC method was compared with other previously established methodologies for the determination of various flavonoids in different samples, as listed in Table 8. A precise observation indicates that the current method showcases similar LOD values and RSD comparable to those reported in prior studies, confirming its efficacy for the quantitative analysis of target analytes across an extensive linear range. Our findings highlight the eco-friendly nature of the proposed method based on hydrophobic DESs, emphasizing their sensitivity and successful performance in extracting and determining target analytes in authentic vinegar samples.

### 2.9. Comprehensive Analysis of the Potential of DES-VALLME Based Programs

As far as we know, flavonoid extraction technologies include alcohol extraction, microwave extraction (MAE), supercritical fluid extraction (SFE), ultrasonic-assisted extraction (UAE), vortex-assisted extraction (VAE) and solid phase extraction (SPE). Among these, supercritical fluid extraction technology is considered a sustainable extraction method, as supercritical fluid can be combined with other conventional non-toxic/low toxic solvents [53,54]. However, the expensive equipment used in SFE hinders its wider application. In contrast, DES-VALLME is an efficient extraction technology that is more economical and simpler to operate than SFE [55,56]. Therefore, DES-VALLME is expected to show great potential in the analysis of various bioactive substances and contribute to the construction of more green and environmentally friendly chemical analysis methods in the future.

## 3. Experimental

### 3.1. Materials and Reagents

Ultrapure water was procured from Hangzhou Wahaha Group Co., Ltd. (Zhejiang, China). Various flavonoid standards were acquired, each adhering to high purity constraints. These included catechin (≥99%), protocatechin (≥98%), rutin (≥99%), hyperoside (≥99%), naringin (≥99%), hesperidin (≥99%), naringenin (≥99%), kaempferol (≥99%) and hesperetin (≥99%), which were obtained from Shanghai Winherb Medical Science Co., Ltd. (Shanghai, China).

A standard reserve solution with a concentration of 1 mg/mL was prepared by accurately weighing 10 mg of each standard flavonoid, which was then dissolved in 10 mL chromatography-grade methanol. This solution served as a ready-to-use stock solution for the experiments. Whenever a standard substance was needed, this stock solution was diluted to form a concentration gradient ranging from 50.00 μg/mL to 0.20 μg/mL.

### 3.2. Instruments and Analytical Conditions

The equipment utilized in this study encompassed an SB-5200DT ultrasonic cleaner supplied by Ningbo Scientz Biotechnology Co., Ltd. (Zhejiang, China) and a high-speed TG16A-WS centrifuge procured from Hunan Saitexiangyi centrifuge instrument Co., Ltd. (Hunan, China). A WH260-R hotplate stirrer was acquired from Wiggens Technology Co., Ltd. (Straubenhardt, Germany).

The chromatographic analyses were carried out using an Agilent 1260 HPLC system (Agilent Technologies, USA). Chromatographic separation was conducted on a C18 column (250 mm × 4.6 mm, 5 µm) sourced from Waters Technologies (Ireland), whose temperature was consistently maintained at 40 °C. The injection volume used for each run was set at 20 μL. The flow rate of the column was standardized to 1.0 mL/min. An analytical wavelength of 280 nm was selected for the detection of flavonoid compounds. The mobile phase comprised a phosphoric acid solution with concentration of 0.1% (Phase A) and acetonitrile (Phase B). The gradient elution procedure was performed as follows: the percentage of Phase A was reduced from 90% to 65% over 0–30 min. Subsequently, at the 30-min mark, the percentage of Phase A was increased from 65% to 90% over a 15-min duration until the 45-min mark. For the final 10 min of the procedure, Phase A was maintained at a constant 90%.

### 3.3. Real Samples Collection

We collected five brands of Shanxi aged vinegar from local supermarket, factories and network. Sample information was as follows: brand C contained C1, C3, C5, C8, C10; brand Y contained Y4, Y6, Y8, Y12, YNJZ, YNM, YNJN, YN1, YN3, YN4, YN5, YN6, YN8, YS3, YS5, YS10; brand W contained W1, W2, W3, W5, W6, WS8, W8, W10; brand D contained DS3, D3, DS5, D5, D8; and brand Z contained Z1, Z3, Z5, Z6, Z8, Z10.

### 3.4. Preparation of Hydrophobic Deep Eutectic Solvent

The DESs were prepared using the tricaprylylmethylammonium chloride as HBA and ethylene glycol, n-caprylic acid, 1,4-butanediol, 2,3-butanediol, 1,3-butanediol as HBD. These components were mixed in specific molar ratios and placed in a centrifuge. The mixture was then heated in a boiling water bath until the liquid became clear and transparent. The resulting DESs were labeled as DES1, DES2, DES3, DES4 and DES5 (Table 9).

### 3.5. Vortex Assisted Liquid–liquid Microextraction Procedure

**Figure 6:**
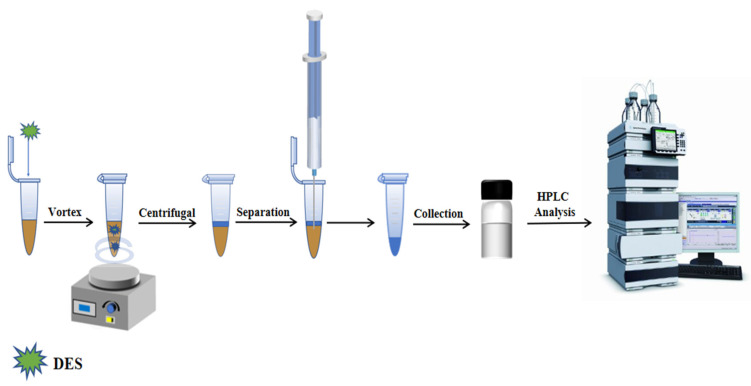
Vortex assisted liquid–liquid microextraction process.

We accurately measured 3.5 mL of the diluted sample solution and transferred it into a 10 mL centrifuge tube. Then, 150 μL DES5 and 0.40 g NaCl were added to the solution. The tube was placed in a vortex mixer for 5 min to reach extraction equilibrium. Afterwards, the tube was centrifuged at 5000 r/min for 10 min. The lower aqueous phase was extracted and discarded using a syringe, while the organic phase was collected using a long needle and passed through a 0.22 μm organic filter membrane. The resulting organic solution was analyzed using HPLC. The VALLME-HPLC procedure is illustrated in Figure 6.

### 3.6. Optimization of Extraction Conditions

In this part, we investigated six factors that affect the extraction effect: the type of DES (DES1, DES2, DES3, DES4, DES5), the molar ratio of HBA to HBD (1:1, 1:2, 1:3, 1:4, 1:5, 1:6, 1:7), the volume of the DES (100 μL, 150 μL, 200 μL, 250 μL, 300 μL), vortex time (2 min, 3 min, 4 min, 5 min, 6 min, 7 min), the amount of NaCl added (0.15 g, 0.20 g, 0.25 g, 0.30 g, 0.35 g, 0.40 g, 0.45 g), and centrifugation time (5 min, 10 min, 15 min, 20 min, 25 min). While investigating one factor, we kept the other five factors constant and conducted three parallel experiments for each group.

## 4. Conclusions

In this study, hydrophobic DES-VALLAME-HPLC was used to determine the quantities of nine flavonoids found in SAV. The analysis utilized a hydrophobic deep eutectic solvent (DES) as an eco-friendly extraction medium during the sample processing phase. Among the five synthesized DESs, DES5 (tricaprylylmethylammonium chloride and 1,4-butanediol, 1:6) had the highest extraction efficiency. This can be attributed to the hydrogen bonding between the target analytes and the solvent. The parameters for the DES-VALLAME-HPLC method were optimized using single factor optimization and BBD design, resulting in good methodological information. The method was subsequently validated validated through matrix-matched calibration, and by evaluating LODs, LOQs and precision. The procedure resulted in satisfactory recoveries (ranging from 85.97% to 108.11%) and low LODs (varying between 0.09–0.18 μg/mL). Furthermore, the greenness of the developed procedure was assessed via AES, GAPI and AGREE, all of which indicated excellent environmental friendliness. The proposed method is fast, cost-effective and sustainable and can be applied to the quality control analysis of flavonoids in SAV without harmful effects on human health or the environment.

## Figures and Tables

**Table 1:** Factors and levels used in the response surface design.

Factors	Levels		
	−1	0	1
The volume of the DES/μL (A)	100	150	200
Vortex time/min (B)	4	5	6
Addition amount of NaCl/g (C)	0.35	0.40	0.45
Centrifugation time/min (D)	5	10	15

**Table 2:** Experimental design and results for response surface design.

	**Catechin**		**Protocatechin**		**Rutin**		**Hyperoside**		**Naringin**	
	**F**	***p***	**F**	***p***	**F**	***p***	**F**	***p***	**F**	***p***
Model	14.84	<0.0001	28.71	<0.0001	18.27	<0.0001	9.94	<0.0001	10.85	<0.0001
A	28.77	<0.0001	35.09	<0.0001	28.89	<0.0001	16.51	0.0012	16.54	0.0012
B	1.42	0.2538	1.64	0.2214	2.43	0.1414	6.61	0.0222	0.85	0.3710
C	0.29	0.6002	7.87	0.0140	2.33	0.1495	3.59	0.0790	0.0024	0.9613
D	1.42	0.2526	5.83	0.0300	1.99	0.1797	0.0001	0.9918	1.13	0.3063
AB	0.29	0.6016	0.31	0.5887	1.61	0.2258	0.010	0.9200	0.051	0.8239
AC	0.57	0.4611	3.45	0.0846	2.00	0.1795	0.32	0.5825	1.80	0.2016
AD	0.062	0.8068	0.56	0.4659	0.010	0.9215	0.11	0.7453	0.31	0.5851
BC	9.28	0.0087	4.27	0.0578	1.76	0.2061	3.65	0.0769	1.21	0.2904
BD	0.068	0.7981	0.083	0.7774	0.0002	0.9895	0.89	0.3611	0.028	0.8695
CD	0.0041	0.9498	1.81	0.2001	1.42	0.2538	0.034	0.8573	0.088	0.7708
A^2^	152.62	<0.0001	316.73	<0.0001	207.56	<0.0001	105.71	<0.0001	46.63	<0.0001
B^2^	17.24	0.0010	30.15	<0.0001	8.98	0.0096	4.82	0.0455	23.93	0.0002
C^2^	10.68	0.0056	1.36	0.2628	0.20	0.6641	0.80	0.3857	21.86	0.0004
D^2^	31.54	<0.0001	46.11	<0.0001	6.46	0.0235	1.55	0.2341	95.55	<0.0001
Lack of fit	0.37	0.9105	1.63	0.3370	2.77	0.1689	0.59	0.7756	3.74	0.1078
R^2^	0.9369		0.9663		0.9481		0.9086		0.9156	
Adjusted R^2^	0.8738		0.9327		0.8962		0.8172		0.8312	
		**Hesperidin**			**Naringenin**		**Kaempferol**		**Hesperetin**	
		**F**	***p***		**F**	***p***	**F**	***p***	**F**	***p***
Model		9.99	<0.0001		11.09	<0.0001	11.45	<0.0001	9.29	<0.0001
A		55.40	<0.0001		71.28	<0.0001	34.18	<0.0001	11.96	0.0038
B		0.38	0.5477		9.54	0.0080	4.39	0.0548	0.77	0.3953
C		0.050	0.8259		0.30	0.5942	3.77	0.0727	16.18	0.0013
D		0.45	0.5124		6.58	0.0225	2.69	0.1232	0.030	0.8644
AB		2.72	0.1214		0.81	0.3833	0.17	0.6877	0.026	0.8748
AC		0.0010	0.9752		0.026	0.8744	1.27	0.2795	0.022	0.8834
AD		0.87	0.3668		1.57	0.2300	19.56	0.0006	0.25	0.6236
BC		2.81	0.1160		3.92	0.0678	0.0082	0.9293	1.69	0.2140
BD		0.028	0.8688		3.00	0.1054	0.49	0.4947	0.041	0.8419
CD		3.72	0.0744		15.23	0.0016	0.019	0.8921	0.14	0.7146
A^2^		35.97	<0.0001		15.81	0.0014	41.27	<0.0001	14.85	0.0018
B^2^		8.16	0.0127		33.89	<0.0001	47.28	<0.0001	3.90	0.0683
C^2^		1.85	0.1957		4.01	0.0651	34.23	<0.0001	10.43	0.0060
D^2^		35.31	<0.0001		4.73	0.0473	0.38	0.5455	93.35	<0.0001
Lack of fit		1.67	0.3290		3.18	0.1383	2.62	0.1831	3.22	0.1356
R^2^		0.9090			0.9173		0.9197		0.9028	
Adjusted R^2^		0.8180			0.8346		0.8393		0.8057	

**Table 3:** The figures of merit of the developed DES-VALLME-HPLC of nine flavonoids.

Analytes	Standard Curve	LR ^a^	R^2 b^	LOD ^c^	LOQ ^d^	EF ^e^	RSD ^f^ (%)	
							Intra-Day	Inter-Day
Catechin	Y = 23.954x − 2.1598	0.2–50	0.9989	0.09	0.30	58	2.90	4.01
Protocatechin	Y = 24.978x − 10.249	0.2–50	0.9990	0.09	0.30	156	2.49	3.04
Rutin	Y = 34.627x + 20.352	0.2–50	0.9968	0.14	0.45	296	2.88	3.72
Hyperoside	Y = 44.449x + 21.951	0.2–50	0.9973	0.12	0.40	54	2.99	4.24
Naringin	Y = 47.103x + 52.2410	0.2–50	0.9946	0.14	0.45	62	3.77	4.53
Hesperidin	Y = 50.146x + 43.7360	0.2–50	0.9944	0.18	0.60	87	2.34	3.11
Naringenin	Y = 144.3x − 15.644	0.2–50	0.9964	0.15	0.50	83	3.30	3.98
Kaempferol	Y = 145.1x − 13.07	0.2–50	0.9990	0.15	0.50	144	3.35	4.30
Hesperetin	Y = 47.795x − 32.546	0.2–50	0.9984	0.12	0.40	43	2.55	4.96

^a^ Linear range (μg/mL). ^b^ Correlation coefficients (R^2^). ^c^ Limit of detection (μg/mL). ^d^ Limit of quantitation (μg/mL). ^e^ Enrichment factor. ^f^ Relative standard deviation.

**Table 4:** The recovery rates of nine kinds of flavonoids in real samples.

Analytes	Sample	Amount Added (μg/mL)	Recovery (%)	Analytes	Sample	Amount Added (μg/mL)	Recovery (%)
Catechin	W3	20	104.97	Hesperidin	W3	20	103.88
	D3	20	103.71		D3	20	98.26
	C2	20	99.54		C2	20	99.34
	YN1	20	102.75		YN1	20	90.73
	Z3	20	97.73		Z3	20	89.10
Protocatechin	W3	20	99.74	Naringenin	W3	20	98.72
	D3	20	107.20		D3	20	96.87
	C2	20	94.99		C2	20	95.86
	YN1	20	98.28		YN1	20	91.33
	Z3	20	108.11		Z3	20	93.28
Rutin	W3	20	93.48	Kaempferol	W3	20	88.19
	D3	20	94.70		D3	20	90.86
	C2	20	96.77		C2	20	86.34
	YN1	20	98.60		YN1	20	87.27
	Z3	20	104.15		Z3	20	85.97
Hyperoside	W3	20	96.19	Hesperetin	W3	20	94.54
	D3	20	91.53		D3	20	93.64
	C2	20	93.33		C2	20	99.31
	YN1	20	97.02		YN1	20	104.32
	Z3	20	101.46		Z3	20	103.22
Naringin	W3	20	93.29				
	D3	20	94.84				
	C2	20	89.10				
	YN1	20	91.84				
	Z3	20	95.61				

**Table 5:** The greenness profile of the proposed method using the eco-scale tool.

Items			PPs
1. Reagent			
Tricaprylylmethylammonium chloride	Amount	<10 mL	1
	Hazard type	Signal word: warning	1
	Hazard amount	1 pictogram	1
Total PPs = 1			
1,4-Butanediol	Amount	<10 mL	1
	Hazard type	Signal word: warning	1
	Hazard amount	1 pictogram	1
Total PPs = 1			
methanol	Amount	<10 mL	1
	Hazard type	Signal word: danger	2
	Hazard amount	3 pictograms	3
Total PPs = 6			
phosphoric acid	Amount	<10 mL	1
	Hazard type	Signal word: danger	2
	Hazard amount	1 pictogram	1
			Total PPs = 2
acetonitrile	Amount	<10 mL	1
	Hazard type	Signal word: danger	2
	Hazard amount	2 pictograms	2
Total PPs = 2			
2. Instruments			
2.1. Energy (kW/h per sample)	HPLC	≤0.1 kWh per sample	0
2.2. Occupational hazard		Emission of vapors and gases to the air	0
3.Waste			
3.1. Waste amount		>10 mL	5
3.2. Waste treatment		No treatment	3
			Total PPs = 8
Total penalty points = 20			
Eco-scale score			100 − 20 = 80

**Table 6:** The greenness profile of the developed method using the GAPI.

GAPI Pictograms		
Sample preparation		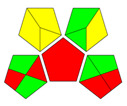
Collection (1)	Off line (red)	
Preservation (2)	None (green)	
Transport (3)	Required (yellow)	
Storage (4)	Simple treatment (yellow)	
Type of method		
Direct or indirect (5)	Extraction required (red)	
Scale of extraction (6)	Micro-extraction (yellow)	
Solvents/reagents used (7)	Green solvents for extraction (yellow)	
Additional treatments (8)	No additional treatment (green)	
Reagent and solvents		
Amount (9)	<10 mL (green)	
Health hazard (10)	NFPA = 1; slightly toxicity(green)	
Safety hazard (11)	NFPA = 3; high flammability (yellow)	
Instrumentation		
Energy (12)	≤0.1 kw h per sample (green)	
Occupational hazard (13)	Hermetic sealing of analytical procedure (green)	
Waste (14)	>10 mL (>10 g)	
Waste treatment (15)	No treatment (red)	

**Table 7:** AGREE results of reported studies for determining flavonoids in SAV.

Reference	Actual Work	[44]	[45]	[46]
Sample Preparation	VALLME	UAE	DLLME	DLLME
Extraction solvent	TOMAC: BDO, 1:6 (150 μL)	60% Ethanol (33.6 mL)	CHCl_3_ (450 μL)	chloroform (150 µL)
Sample (g/mL)	3.5 mL	2 g	10 mL	10 g
Detection	HPLC	UV	UHPLC–UV analysis	LC-DAD-ESI-ToFMS
AGREE plot	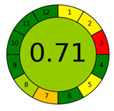	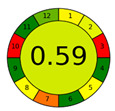	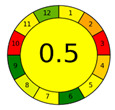	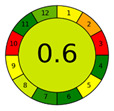

**Table 8:** Comparison of the developed method with other methods for the analysis of the flavonoids.

Method	Sample	Analytes	Analytical Technique	LR	LOD	RSD (%)		Ref.
						Intra-Day	Inter-Day	
DES-HLLME	Scutellariae Radix	6 kinds of flavonoids	HPLC-UV	0.0022–8.65 mg/L	≤8.0 g/L	0.1–7.8	0.2–9.2	[47]
Ultrasonic extraction	Dalbergia odorifera	17 kinds of flavonoids	UHPLC-MS/MS	0.516–5652 ng/mL	0.085–1.790 g/mL	0.45–3.51	1.26–4.94	[48]
SPE	Chinese wolfberry, orange juice and wine samples	4 kinds of flavonoids	HPLC	1–500 ng/mL	0.15–0.41 ng/mL	2.64–4.56	3.64–4.20	[49]
UAE	Hawk tea	3 kinds of flavonoids	UPLC-DAD	0.36–880 μg/mL	0.086–0.308 μg/mL	1.16–4.18	1.63–4.26	[50]
MSPE-DES	orange, apple, onion and green tea	4 kinds of flavonoids	HPLC-UV	0.03–0.14 μg/L	0.1–0.5 ng/mL	≤5.4	≤5.6	[51]
CF-ASME	Ginkgo biloba and Platycladus orientalis	5 kinds of flavonoids	HPLC-UV	0.01–5 μg/mL	0.5–30 ng/mL	1.8–12.6	3.3–12.8	[52]
DES-VALLME	Shanxi aged vinegar	9 kinds of flavonoids	HPLC	0.20–50.00 μg/mL	0.09–0.18 μg/mL	2.34–3.77	3.04–4.96	This work

**Table 9:** Types of different DES.

DES	HBA	HBD	Molar Ratio
DES1	Tricaprylylmethylammonium chloride	n-Caprylic acid	1:2
DES2	Tricaprylylmethylammonium chloride	Ethylene glycol	1:3
DES3	Tricaprylylmethylammonium chloride	2,3-Butanediol	1:2
DES4	Tricaprylylmethylammonium chloride	1,3-Butanediol	1:3
DES5	Tricaprylylmethylammonium chloride	1,4-Butanediol	1:6

## Data Availability

All data, tables, and figures are originals.

## Sample Availability

Samples of the compounds are not available from the authors.
